# Time-Frequency Delta Activity to Social Feedback Demonstrates Differential Associations With Depression and Social Anxiety Symptoms

**DOI:** 10.3389/fnbeh.2019.00189

**Published:** 2019-08-27

**Authors:** Jingwen Jin, Amri Sabharwal, Zachary P. Infantolino, Johanna M. Jarcho, Brady D. Nelson

**Affiliations:** ^1^Department of Psychology, Stony Brook University, Stony Brook, NY, United States; ^2^Department of Psychology, Temple University, Philadelphia, PA, United States

**Keywords:** time-frequency, delta, theta, social feedback processing, social anxiety, depression

## Abstract

Social feedback is highly salient and particularly relevant when investigating the pathophysiology of depression and social anxiety. A bourgeoning body of research has demonstrated an association between reward-related delta activity and psychopathology. However, a critical limitation is that these findings are derived from neural responses to *monetary* feedback, and time-frequency representation of social feedback remains unexplored. In addition, no study has isolated the differential/unique associations of positive valence and the intrinsic rewarding experience of being correct with reward-related neural activity. In the present study, 204 participants underwent electroencephalography (EEG) while they completed a novel paradigm comprised of monetary and social feedback tasks that were matched in trial structure, timing, and feedback stimuli. For each task, participants were instructed to correctly identify one of two doors that would provide positive feedback (monetary win behind the door) or one of two peers who would provide positive feedback (social like); or to correctly identify the door or peer that would provide negative feedback (money loss behind the door/social dislike). A principal component analysis (PCA) was conducted on the time-frequency data and revealed two factors in the delta and one factor in the theta frequency ranges. Results indicated that the lower-frequency delta factor (delta-low) was greater to correct vs. incorrect feedback, more so for social vs. monetary tasks, while the higher-frequency delta factor (delta-high) was greater to correct vs. incorrect feedback for social like, social dislike, and monetary win tasks, but not the monetary loss task. In contrast, the theta factor was greater to incorrect relative to correct feedback in negative valence (lose money/social dislike) but not positive valence (win money/social like) tasks. Furthermore, greater delta-high activity for social feedback was associated with greater social anxiety symptoms, whereas lesser delta-high activity for social feedback was associated with greater depressive symptoms. Finally, greater theta activity to monetary feedback was associated with greater depressive symptoms. The present study provides novel evidence demonstrating unique social vs. monetary feedback-related delta and theta activity, and differential associations between delta activity with depression and social anxiety symptoms. These findings highlight the importance of investigating feedback-related neural responses in the social domain.

## Introduction

Understanding how humans process salient feedback is central not only to economic theories of monetary decision-making (Bernoulli, 1954; Tversky and Kahneman, 1992; Von Neumann and Morgenstern, 2007) but also to theories of social decision-making (Homans, 1958; Sanfey, 2007). Adaptive behavioral changes often rely on successful processing of outcome feedback (Everitt and Robbins, 2005; Wrase et al., 2007). Failure to use feedback to flexibly update decision-making strategies has been linked to various mental disorders, such as depression (Cella et al., 2010) and anxiety disorders (Hartley and Phelps, 2012; Phelps et al., 2014). While responses to positive and negative feedback in monetary (win and loss) and social (being accepted/liked and rejected/disliked) domains are both important in daily life, it remains unclear whether the two domains share the same or have unique neural mechanisms. It is also unknown whether neural responses to monetary and social feedback demonstrate common or differential relationships with depression and anxiety symptoms.

Neuroimaging studies comparing monetary and social feedback have demonstrated that the two domains share overlapping neural circuitry, including the striatum and prefrontal regions (Izuma et al., 2008; Lin et al., 2012; Hausler et al., 2015). On the other hand, accumulating evidence also suggests that monetary and social feedback elicit unique neural responses (Rademacher et al., 2010; Chan et al., 2016). For example, using the monetary incentive delay task, one study found that while monetary reward was associated with thalamic activity, social reward was associated with amygdala activity (Rademacher et al., 2010).

Parallel to these neuroimaging studies, event-related potential (ERP) research has identified the reward positivity (RewP), a positive-going component that peaks approximately 250–350 ms following monetary win feedback that is absent or reduced to monetary loss feedback (Holroyd et al., 2008; Foti et al., 2011; Novak and Foti, 2015; Proudfit, 2015). The RewP has been primarily examined in the context of monetary feedback, but more recent studies have shown that it can also be elicited by positive social feedback (e.g., social acceptance; Kujawa et al., 2014; van der Veen et al., 2016). When directly compared within the same participants, one study found a higher RewP to monetary vs. social reward in emerging adults but not in early adolescents, and the monetary and social RewPs were not correlated across the two groups (Ethridge et al., 2017). However, the social paradigm used in this study had important differences compared with the monetary task. For instance, the paradigm required the participants to decide on accepting or rejecting simulated co-players before receiving acceptance/rejection feedback from the same co-players. Additionally, there were timing differences in trial structure (e.g., the social task contained an additional variable delay between making a choice and receiving feedback). A more recent study that matched the designs of the monetary and social tasks found that the RewP to monetary and social feedback was of comparable magnitude and positively correlated, although only the RewP to social feedback was associated with depressive symptoms (Distefano et al., 2018). Taken together, the current literature suggests that when the paradigms are closely matched, monetary and social feedback may elicit overlapping neural responses. On the other hand, feedback from the two domains may exhibit unique and potentially dissociable relationships with particular forms of psychopathology, such as depression and social anxiety.

In addition to monetary and social paradigm differences, the task designs of previous studies often confounded the positive valence of outcome (i.e., monetary win and being socially accepted) with the intrinsic reward of being correct (i.e., the chosen option yielding win/acceptance feedback). One exception was a recent investigation that examined time-frequency indices in response to monetary win vs. loss and correct vs. incorrect feedback (Bernat et al., 2015). In this study, delta activity was higher both for positive valence (i.e., win) compared to negative valence (i.e., loss) and being correct compared to incorrect, and theta activity was higher for negative valence compared to positive valence, but not incorrect vs. correct outcomes (Bernat et al., 2015). However, the correct/incorrect outcome was dependent on valence such that correct (in contrast to incorrect) indicated a larger win or smaller loss, and therefore was secondary to valence. In sum, no study has investigated time-frequency activity to these two dimensions simultaneously as primary feedback attributes.

Previous studies examining electrocortical responses to win and loss feedback have largely focused on time-domain ERPs. While this line of research has yielded largely consistent findings (Bernat et al., 2015; Proudfit, 2015), there are some notable limitations to this analytic approach. Time-domain ERPs consist of multiple temporally-overlapping components that are often characterized by different frequency profiles (Bernat et al., 2005; Dien, 2010b; Foti et al., 2015). Time-frequency based representation of the signal can help elucidate distinct neural processes that occur at different frequency bands (e.g., delta vs. theta activity) that are otherwise embedded in the time-domain data (Spencer et al., 2001). Furthermore, time-frequency analysis of single-trial data allows researchers to identify non-phase locked aspects of the neural response that might be attenuated or absent in the time-domain signal due to the common practice of trial averaging (Bernat et al., 2005; Cohen et al., 2007; Cohen, 2014). Multiple investigations that conducted time-frequency analysis have found that the neural response in the time range of the RewP shows greater delta activity to monetary win feedback and greater theta activity to monetary loss feedback (Bernat et al., 2011, 2015; Foti et al., 2015). However, no study has investigated the time-frequency activity in the context of social feedback and compared that activity to monetary feedback. Given the unique information time-frequency based representation can provide, it is important to examine whether the delta and theta activities are also present for social feedback.

Examining distinct time-frequency indices in response to monetary and social feedback may also help reveal any differential neural correlates of depression and anxiety. Theoretical and empirical research has suggested that depression is associated with a blunted neural response to monetary win and an enhanced neural response to monetary loss (Henriques and Davidson, 2000; Eshel and Roiser, 2010; Kujawa et al., 2014; Luking et al., 2016). In addition, a blunted RewP to monetary win (compared to loss) has been shown to prospectively predict depressive symptoms and syndromes (Bress et al., 2013; Nelson et al., 2016). Two recent studies using social reward tasks also demonstrated a blunted RewP in association with depression (Kujawa et al., 2014; Distefano et al., 2018). To date, only a small number of studies have examined time-frequency neural activity to monetary feedback in relation to depression. One study found that blunted delta activity to monetary reward was associated with greater depression, anxiety, and stress (Foti et al., 2015). Conversely, a separate study of adolescent girls found that depression was associated with higher loss-related theta activity, but there were no group differences in reward-related delta activity (Webb et al., 2017). Finally, a recent investigation of adolescent girls found that blunted delta activity to monetary reward prospectively predicted first-onset depression, independent of the time-domain RewP (Nelson et al., 2018). Together, this nascent literature suggests that depression might be associated with an aberrant neural response in particular frequency bands. However, no study has examined time-frequency activity to social feedback in relation to depressive symptoms.

Even less is known about the relationship between the neural response to monetary and social feedback and social anxiety. One study using a child sample found that a greater RewP was associated with higher social anxiety symptoms even after controlling for depressive symptoms (Kessel et al., 2015). An important limitation of the current literature is that the neural response to feedback is often examined using monetary tasks, and there is a lack of research examining the ERP response to social feedback in relation to social anxiety. As the hallmark of social anxiety is the fear of social evaluation (American Psychiatric Association, 2013), it is possible that the association between the neural response to feedback and social anxiety is more sensitive to social compared to non-social information. However, no study has examined the time-frequency indices of neural response to social compared to monetary feedback in relation to social anxiety symptoms.

To address these issues, the current study utilized a novel paradigm that carefully matched the trial structure, timing, and visual presentation of feedback stimuli. This design permitted the comparison of participants’ neural response to feedback indicating monetary win, monetary loss, social acceptance (i.e., being liked), and social rejection (i.e., being dislike). Furthermore, the tasks were designed to tease apart the effects of feedback domain (monetary vs. social), valence (positive vs. negative), and outcome (correct vs. incorrect). In a large sample of young adults, we employed time-frequency analysis to examine delta and theta activity to feedback across both monetary and social domains. In addition, we investigated relations between these time-frequency indices and individual differences in depression and social anxiety symptoms. We hypothesized that: (1) for both the monetary and social domains, there would be higher delta activity to positive and/or correct feedback and higher theta activity to negative and/or incorrect feedback; and (2) blunted delta activity to positive feedback (across both monetary and social domains) would be associated with more severe depressive symptoms. Due to the exploratory nature of the remaining analyses, we did not have other specific hypotheses for time-frequency activity to social feedback or social anxiety symptoms.

## Materials and Methods

### Participants

Two hundred and five participants were recruited, with one excluded due to not completing the experiment. The final sample included 204 participants (*M* = 19.92 years old, *SD* = 2.50), who were 63.7% female, racially/ethnically diverse (45.1% Asians, 5.9% Black, 26.5% Caucasian, 10.8% Latino, and 11.8% “Other”), and participated for course credit. All participants gave informed consent and the study was approved by the Stony Brook University Institutional Review Board.

### Measures

Participants completed the Inventory of Depression and Anxiety Symptoms—Expanded Version (IDAS-II; Watson et al., 2007, 2012). IDAS-II is a factor analytically-derived self-administered questionnaire that assesses symptomatology of mood and anxiety disorders in the past 2 weeks using a Likert scale ranging from 1 (*not at all*) to 5 (*extremely*). IDAS-II has demonstrated excellent psychometric properties across various populations, including college students, community, and patient samples (Watson et al., 2012). The current study focused on the 10-item dysphoria scale (*M* = 19.47, *SD* = 7.52, Cronbach’s α = 0.88), which is the most discriminant symptom dimension of major depressive disorder, and the 6-item social anxiety scale (*M* = 10.95, *SD* = 5.22, Cronbach’s α = 0.86).

### Stimuli

The social feedback task stimuli were identical to a previous investigation (Distefano et al., 2018) and consisted of 120 images of age-matched peers (60 females) compiled from multiple sources [e.g., National Institute of Mental Health’s Child Emotional Faces picture set (Egger et al., 2011), internet databases of non-copyrighted images, and photographs of college-aged individuals]. Variability in the appearance of the social stimuli was necessary in order to corroborate task deception, which suggested participants were being evaluated by actual peers. All images were cropped to a standardized size (3.5" width × 4.5" height), and occupied approximately 8° of visual space horizontally and 10° vertically for participants seated approximately 24" from the monitor. Each trial slide contained a pair of either male peers or female peers (60 pairs of male faces and 60 pairs of female faces), pictured from their shoulders up, with a positive facial expression and a solid background.

### Procedure

At the beginning of the experimental session, participants were told that they would complete a social evaluation study with peers at different universities across the United States. Participants were asked to provide a digital photo of themselves that was purportedly uploaded to a study database. Participants believed that once this photograph was uploaded, peers would receive a text message on their cell phone asking them to view the photo and indicate whether they thought they would “like” or “dislike” the participant. Participants were told that later in the experimental session, after enough time had elapsed for the purported peers to have rated their photo, they would be asked to guess which peers “liked” and “disliked” them. Participants were also told that they would be completing monetary guessing tasks. Next, participants completed self-report questionnaires while an electroencephalography (EEG) cap was applied to their head. Finally, participants completed the monetary and social feedback tasks in a counterbalanced order.

#### Monetary and Social Feedback Tasks

The monetary and social feedback tasks were administered using Presentation software (Neurobehavioral Systems, Inc., Albany, CA, USA) and were modified variants of previously established tasks (Proudfit, 2015; Distefano et al., 2018). Overall, there were four total tasks (monetary win, monetary loss, social like, and social dislike) that were presented in a counterbalanced order.

**Figure 1:**
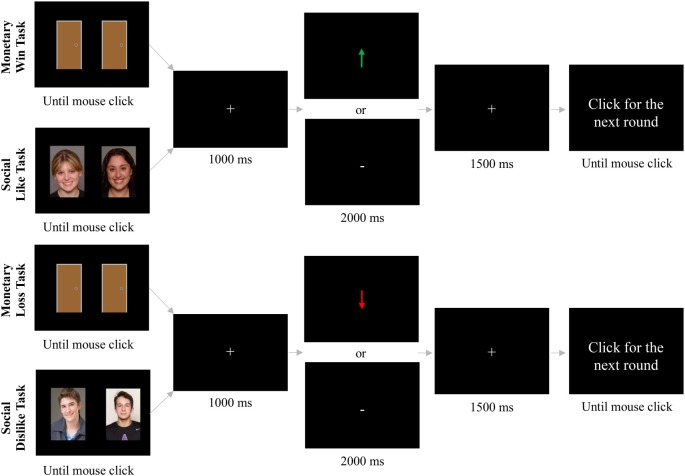
Schematic for monetary win, monetary loss, social like, and social dislike tasks.

Figure 1 displays the overall task schematic. In the monetary win task, each trial began with the presentation of two identical doors. Participants were told there were three possible scenarios for each trial: (1) both doors contained a $0.25 monetary win; (2) one door contained a $0.25 monetary win while the other door resulted in a break-even outcome (i.e., neither win nor lose); or (3) both doors resulted in a break-even outcome. These instructions ensured that the feedback the participant received would only be informative about the door they chose and not the door they *did not* choose. For example, if a participant chose a door and received feedback indicating a break-even result, the other door could have been a win door [consistent with trial scenario (2) above] or it could have been a break-even door [consistent with trial scenario (3) above]. Conversely, if a participant chose a door and received feedback indicating a win result, the other door could have been a win door [consistent with trial scenario (1) above] or it could have been a break-even door [consistent with trial scenario (2) above]. Participants were told that the goal of these trials was to try and guess which door contained the monetary win. The image of the doors was presented until the participant made a selection. After stimulus offset, a fixation cross (+) was presented for 1,000 ms, and then feedback was presented on the screen for 2,000 ms. Correct selection of the monetary win door resulted in a $0.25 monetary win, indicated by a green arrow pointing upward (↑). Incorrect selection of the break-even door resulted in no monetary win, indicated by a white horizontal dash (–). In actuality, feedback was pre-programmed to generate an equal number of win and break-even trials. The feedback stimulus was followed by a fixation cross presented for 1,500 ms, immediately followed by the message “Click for next round.” This prompt remained on the screen until the participant responded with a button press to initiate the next trial. The task consisted of 30 total trials (15 of each outcome).

In the monetary loss trials, trial structure and timing was identical to the monetary win trials, but participants were told there were three possible situations for each trial: (1) both doors contained a $0.25 monetary loss; (2) one door contained a $0.25 monetary loss while the other door resulted in a break-even outcome (i.e., neither win nor lose); or (3) both doors resulted in a break-even outcome. Participants were told that the goal of these trials was to try and guess which door contained the monetary loss. Correct selection of the monetary loss door resulted in a $0.25 monetary loss, indicated by a red arrow pointing downward (↓). Incorrect selection of the break-even door resulted in no monetary loss, indicated by a white horizontal dash (–). All participants were told that they would start with a pot of $5. Given that there were equal number of wins and losses, they were paid $5 at the end of the experiment.

The social like and dislike tasks were identical to the monetary win and loss tasks, respectively, except pictures of gender-matched peers (i.e., two male faces or two female faces) were presented instead of doors. There was an equal number of trials with male and female peers across the social like and social dislike tasks (30 each, 60 total). In the social like trials, participants were told that there were three possible situations for each trial: (1) both people said they would like the participant; (2) one person said they would like the participant while the other person never rated the participant; or (3) neither person rated the participant. Participants were told that the goal of these trials was to try and guess which person said they would like the participant. Correct selection of the person who said they would like the participant was indicated by a green arrow pointing upward (↑). Incorrect selection of the person who never rated the participant was indicated by a white horizontal dash (–).

In the social dislike trials, participants were told there were three possible situations for each trial: (1) both people said they would dislike the participant; (2) one person said they would dislike the participant while the other person never rated the participant; or (3) neither person rated the participant. Participants were told that the goal of these trials was to try and guess which person said they would dislike the participant. Correct selection of the person who said they would dislike the participant was indicated by a red arrow pointing downward (↓). Incorrect selection of the person who never rated the participant was indicated by a white horizontal dash (–). Participants took about 5–7 min for each task.

#### EEG Recording and Processing

Continuous EEG was recorded using an elastic cap with 34 electrode sites placed according to the 10/20 system. Electrooculogram (EOG) was recorded using four additional facial electrodes: two placed approximately 1 cm outside of the right and left eyes and two placed approximately 1 cm above and below the right eye. All electrodes were sintered Ag/AgCl electrodes. Data were recorded using the ActiveTwo system (BioSemi, Amsterdam, Netherlands). The EEG was digitized with a sampling rate of 1,024 Hz using a low-pass fifth-order sinc filter with a half-power cut-off of 204.8 Hz. A common mode sense active electrode producing a monopolar (non-differential) channel was used as recording reference.

Offline data processing was conducted using EEGLAB toolbox version 13.6.5b (Delorme and Makeig, 2004) and customized MATLAB scripts (The MathWorks, Inc., Natick, MA, USA). EEG data were first re-referenced to the average of the left and right mastoids, high-pass filtered (0.01 Hz) to remove baseline drift, and segmented into single-trial epochs (−3,000, +3,000 ms) around the feedback onset. Epochs containing artifacts were identified and rejected using Fully Automated Statistical Thresholding for EEG Artifact Rejection (Nolan et al., 2010). Consistent with published guidelines (Nolan et al., 2010), the decision to reject epochs was based on three parameters: the amplitude range of the epoch, the deviation between the epoch and the channel average, and the variance within the epoch. The parameters were converted to *z*-scores and epochs with an absolute *z*-score greater than three were identified and rejected. Eye blinks artifacts were then removed using independent component analyses. The number of trials went into the time-frequency analyses for each condition were: *M* = 14.77 (*SD* = 0.77) for monetary loss correct, *M* = 14.78 (*SD* = 0.92) for monetary loss incorrect, *M* = 14.82 (*SD* = 0.77) for monetary win correct, *M* = 14.86 (*SD* = 0.52) for monetary win incorrect, *M* = 14.80 (*SD* = 0.88) for social dislike correct, *M* = 14.72 (*SD* = 1.05) for social dislike incorrect, *M* = 14.73 (*SD* = 1.09) for social like correct, *M* = 14.79 (*SD* = 0.97) for social like incorrect.

In order to retain phase and non-phase locked neural responses (Cohen, 2014; Luck, 2014), single-trial epochs for each electrode were then decomposed into their time-frequency representation using Morlet wavelets. Specifically, the power spectrum of the epochs was multiplied by the power spectrum of a set of complex Morlet wavelets that increased by 33 logarithmic steps from 1 to 13 Hz. The frequency band-specific power at each time point was calculated by squaring the absolute value of the complex signal. A decibel transformation was used to normalize the power. Specifically, we took the logarithm of the ratio of post-feedback power divided by the average baseline (−200 to 0 ms) power for each frequency.

**Figure 2:**
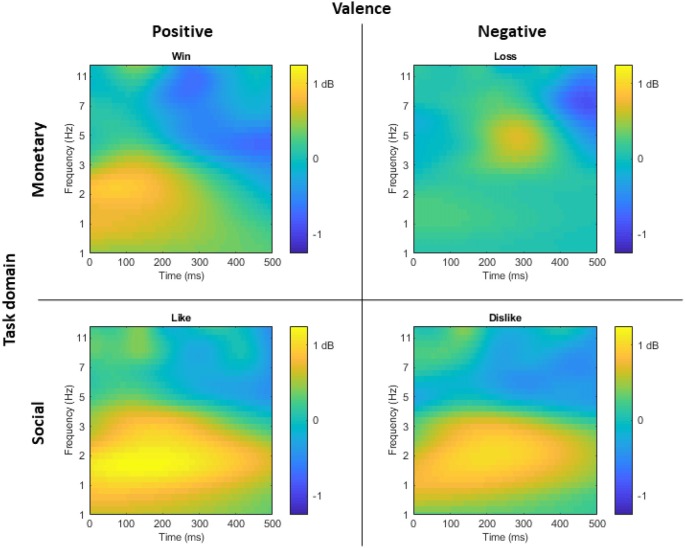
For visual display of time-frequency representation of the task-effect, time-frequency plots displaying the difference at FCz between correct and incorrect trials for monetary win, monetary loss, social like, and social dislike tasks, respectively. Yellow indicates greater activity for correct compared to incorrect trials, while blue indicates greater activity for incorrect compared to correct trials.

**Figure 3:**
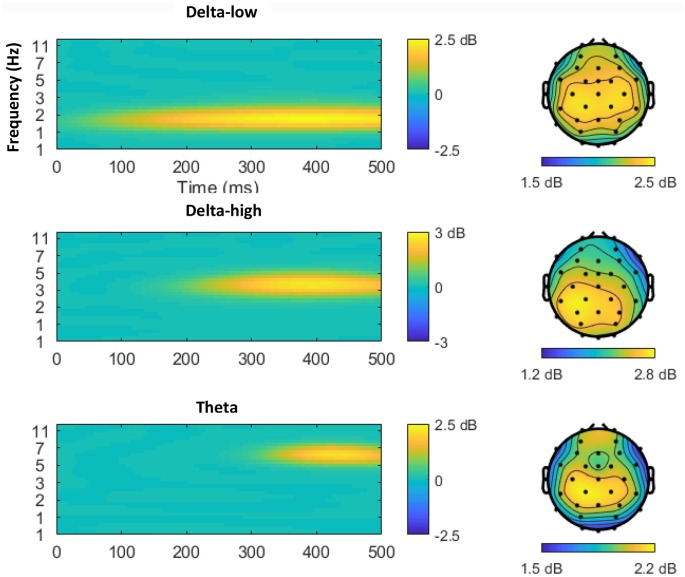
Time-frequency-spatial principal component analysis (PCA) revealed three factors in the delta to theta frequency bands within the time range of the reward positivity (RewP). From top to bottom, the three factors are ordered by the percentage of variance explained from largest to smallest. The left column depicts the time-frequency power spectrum of each factor, with yellow indicating higher power. The right column depicts the scalp distribution of the corresponding factor.

Figure 2 displays the time-frequency plots for all four tasks. Following established guidelines (Bernat et al., 2005), a two-step principal component analysis (PCA) was conducted to better isolate distinct neural responses. Time-frequency surfaces for the 0–500 ms post-feedback segment were vectorized and entered into PCA Toolkit version 2.52 (Dien, 2010a) to conduct a PCA using the time-frequency vectors as variables and the participants, outcomes (correct and incorrect), and tasks (monetary win, monetary loss, social like, social dislike) as observations. Varimax rotation was applied and 55 factors were extracted based on the resulting Scree plot (Cattell, 1966). A spatial PCA was then conducted using an Infomax rotation and four factors were extracted based on the resulting Scree plot (Cattell, 1966). The two-step PCA resulted in 220 temporal-frequency spatial factors in total. With a cut-off of at least 0.5% of the variance explained, 33 factors emerged, accounting for 65.4% variance altogether. Next, we identified PCA factors for data analysis based on a two-step visual inspection approach. First, we organized the factors in order from the most to the least variance accounted for, and we ignored all factors that accounted for <1% of the variance. Second, we only examined factors that overlapped with the delta or theta frequency ranges and contained spatial distributions that centered around frontal and parietal regions. As shown in Figure 3, this visual inspection procedure revealed three factors that accounted for the most variance and resembled the expected delta (two delta factors TF1SF1 and TF2SF1) and theta (one theta factor TF4SF1) activity. The delta factor TF1SF1 centered on the lower frequency range (delta-low) and accounted for 10.38% variance, while delta factor TF2SF1 centered on higher frequency range (delta-high) and accounted for 7.51% variance. The theta factor accounted for 4.97% variance.

#### Data Analyses

We conducted a 2 (Domain: monetary vs. social) × 2 (Valence: positive vs. negative) × 2 (Outcome: correct vs. incorrect) repeated-measures analysis of variance (rmANOVA). Separate analyses were conducted for each PCA factor. We also conducted a 2 (Domain: monetary vs. social) × 2 (Valence: positive vs. negative) × 2 (Outcome: correct vs. incorrect) mixed-measures analysis of covariance (ANCOVA), with dysphoria and social anxiety symptoms entered as simultaneous covariates. When dysphoria and/or social anxiety symptoms were associated with PCA factors, linear regression was conducted to compute the residual scores for one symptom dimension independent of the other symptom dimension. These residual scores were then used to further investigate the relationships.

We also conducted a series of analyses examining the potential effects of domain order. For each of the Domain × Valence × Outcome rmANOVAs, we entered Domain Order (monetary first vs. social first) as a between-subjects factor. If significant interactions were identified for the Domain Order variable, we would follow up with additional ANCOVA analyses for depression and social anxiety symptoms, including the Domain Order variable as an additional between-subject covariate. All statistical analyses were conducted in IBM SPSS Statistics, Version 25.0 (Armonk, NY, USA).

## Results

### Monetary and Social Tasks

**Figure 4:**
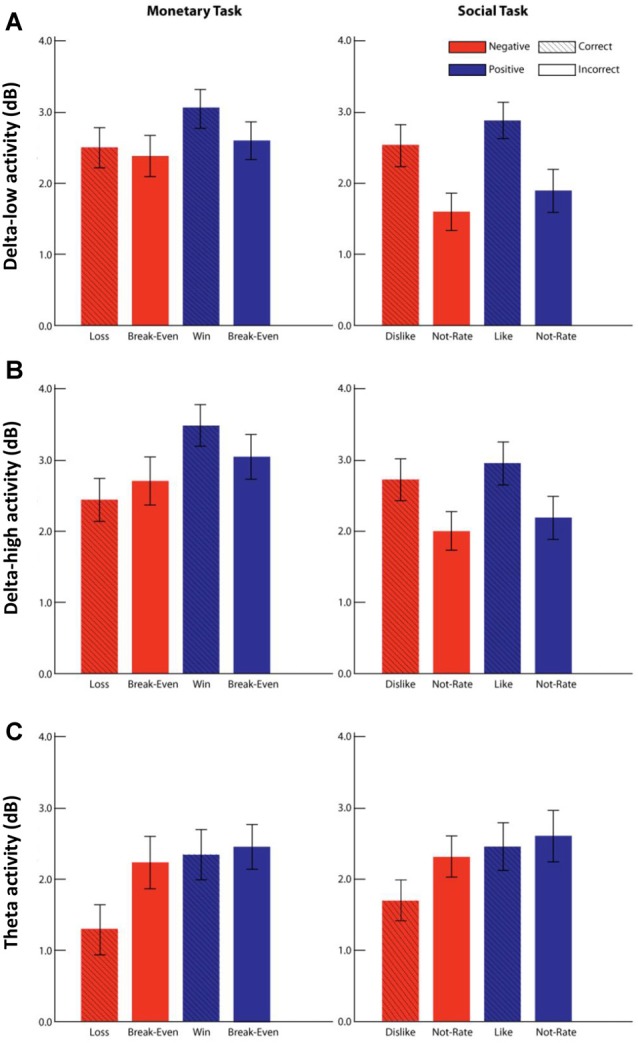
Delta and theta activity during the monetary and social tasks. Panels (**A**–**C**) represent the results for each factor: delta-low (TF1SF1; **A**), delta-high (TF2SF2; **B**), and theta (TF4SF1; **C**). Mean activity for each factor in each condition is shown for monetary (left column) and social (right column) tasks. Error bars represents ± 95% confidence interval. Negative valence is represented in red, and positive valence is represented blue. Correct and incorrect outcomes are differentiated by presence and absence of the line pattern.

Descriptive statistics for the PCA factors and symptom measures were reported in Table 1. For the delta-low factor (Figure 4A), results indicated a main effect of domain with greater delta activity for monetary vs. social feedback, *F*_(1,203)_ = 15.73, *p* < 0.001, $\eta_{\text{p}}^{2}$ = 0.07, a main effect of valence with greater delta activity for positive vs. negative feedback, *F*_(1,203)_ = 17.71, *p* < 0.001, $\eta_{\text{p}}^{2}$ = 0.08, and a main effect of outcome with greater delta activity for correct vs. incorrect feedback, *F*_(1,203)_ = 51.48, *p* < 0.001, $\eta_{\text{p}}^{2}$ = 0.20. There was also a Domain × Outcome interaction *F*_(1,203)_ = 23.95, *p* < 0.001, $\eta_{\text{p}}^{2}$ = 0.11. Simple-effect analyses indicated that delta activity was greater for correct compared to incorrect feedback for both monetary (mean difference = 0.29, *p* < 0.01) and social (mean difference = 0.95, *p* < 0.001) tasks, but this increase was greater for the social compared to monetary task.

**Table 1:** Descriptive statistics.

PCA factors (*N* = 204)			
Factor TF1SF1		Mean	SD
Monetary loss	Correct	2.51	2.01
	Incorrect	2.39	2.09
Monetary win	Correct	3.06	1.91
	Incorrect	2.60	1.86
Social dislike	Correct	2.54	2.07
	Incorrect	1.60	1.90
Social like	Correct	2.88	1.88
	Incorrect	1.90	2.14
**Factor TF2SF1**	
Monetary loss	Correct	2.45	2.18
	Incorrect	2.72	2.44
Monetary win	Correct	3.50	2.12
	Incorrect	3.05	2.29
Social dislike	Correct	2.73	2.11
	Incorrect	2.02	1.95
Social like	Correct	2.96	2.18
	Incorrect	2.20	2.20
**Factor TF4SF1**	
Monetary loss	Correct	1.30	2.47
	Incorrect	2.23	2.65
Monetary win	Correct	2.34	2.53
	Incorrect	2.45	2.31
Social dislike	Correct	1.70	2.09
	Incorrect	2.31	2.10
Social like	Correct	2.45	2.41
	Incorrect	2.61	2.61
**Depression and Social**			
**Anxiety Measures (*N* = 204)**			
Dysphoria	19.47	7.52	
Social anxiety	10.95	5.22	
Pearson’s correlation coefficient	0.664	*p* < 0.001	

For the delta-high factor (Figure 4B), results indicated a main effect of domain with greater delta activity for monetary vs. social feedback, *F*_(1,203)_ = 18.67, *p* < 0.001, $\eta_{\text{p}}^{2}$ = 0.08, a main effect of valence with greater delta activity for positive vs. negative feedback, *F*_(1,203)_ = 23.30, *p* < 0.001, $\eta_{\text{p}}^{2}$ = 0.10, and a main effect of outcome with greater delta activity for correct vs. incorrect feedback, *F*_(1,203)_ = 28.90, *p* < 0.001, $\eta_{\text{p}}^{2}$ = 0.13. There were also Domain × Valence, *F*_(1,203)_ = 7.22, *p* < 0.01, $\eta_{\text{p}}^{2}$ = 0.03, Domain × Outcome, *F*_(1,203)_ = 29.27, *p* < 0.001, $\eta_{\text{p}}^{2}$ = 0.13, and Valence × Outcome interactions, *F*_(1,203)_ = 6.84, *p* = 0.05, $\eta_{\text{p}}^{2}$ = 0.03, which were qualified by a Domain × Valence × Outcome interaction, *F*_(1,203)_ = 6.36, *p* < 0.05, $\eta_{\text{p}}^{2}$ = 0.03. To follow-up the three-way interaction, we conducted separate Valence × Outcome rmANOVAs for monetary and social tasks. Simple-effect analyses indicated that for the monetary tasks, delta activity was greater for correct feedback compared to incorrect feedback for positive valence (i.e., win) trials (mean difference = 0.45, *p* < 0.01), but not for negative valence (i.e., loss) trials (mean difference = −0.27, *ns*). For the social tasks, delta activity was greater for correct compared to incorrect feedback for both positive valence (i.e., like) trials (mean difference = 0.76, *p* < 0.001) and negative valence (i.e., dislike) trials (mean difference = 0.72, *p* < 0.001).

For the theta factor (Figure 4C), results indicated a main effect of outcome with greater theta activity for incorrect vs. correct feedback, *F*_(1,203)_ = 26.72, *p* < 0.001, $\eta_{\text{p}}^{2}$ = 0.12, and a main effect of valence with greater theta activity for positive vs. negative valence trials, *F*_(1,203)_ = 30.76, *p* < 0.001, $\eta_{\text{p}}^{2}$ = 0.13. There was also a Valence × Outcome interaction, *F*_(1,203)_ = 16.37, *p* < 0.001, $\eta_{\text{p}}^{2}$ = 0.08). Simple-effect analyses indicated that theta activity was greater for incorrect vs. correct feedback for negative valence trials (mean difference = 0.77, *p* < 0.001), but not positive valence trials (mean difference = 0.13, *ns*).

### Dysphoria and Social Anxiety Symptoms

**Figure 5:**
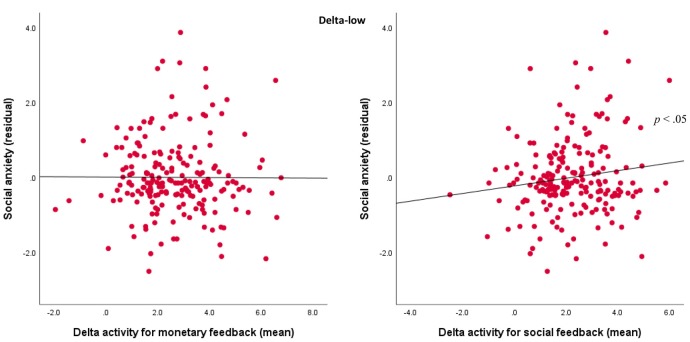
Associations between TF1SF1 (delta-low activity) for social and monetary feedback and symptoms. Scatterplots displaying the associations between social anxiety residuals with delta-low activity for mean monetary feedback averaged across both valence and outcome (left) and mean social feedback averaged across both valence and outcome (right).

For the delta-low factor, results indicated a Domain × Social Anxiety interaction, *F*_(1,201)_ = 4.02, *p* < 0.05, $\eta_{\text{p}}^{2}$ = 0.02. For the follow-up analyses, delta-low activity was averaged across valence and outcome for the monetary and social tasks. As shown in Figure 5, follow-up Pearson’s correlations indicated that more severe social anxiety symptoms were associated with greater delta activity for social feedback (*r* = 0.15, *p* < 0.05), but not for monetary feedback (*r* = 0.05, *ns*).

**Figure 6:**
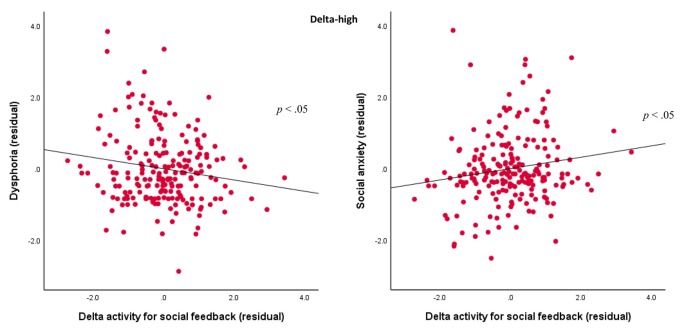
Associations between TF2SF1 (delta-high activity) for social feedback and symptoms. Scatterplots displaying the associations between delta-high activity for social feedback (difference between correct and incorrect trials) residual with dysphoria (left) and social anxiety (right) residuals.

For the delta-high factor, results indicated Domain × Outcome × Dysphoria, *F*_(1,201)_ = 4.24, *p* < 0.05, $\eta_{\text{p}}^{2}$ = 0.02, and Domain × Outcome × Social Anxiety interactions, *F*_(1,201)_ = 6.59, *p* < 0.05, $\eta_{\text{p}}^{2}$ = 0.03. In order to examine these associations, we first averaged delta-high activity values across the positive and negative valence for each domain and outcome combination. Next, we created separate difference scores for correct and incorrect outcomes (i.e., correct-incorrect) for the monetary and social tasks. Finally, in order to examine the variance explained by domain-specific responses, we computed two residual scores to quantify delta-high activity for the monetary (independent of the social difference score) and social (independent of the monetary difference score) tasks. We also calculated residuals for dysphoria (independent of social anxiety) and social anxiety (independent of dysphoria), and we conducted Pearson’s correlations between the two delta-high activity residuals and the two symptom residuals. As shown in Figure 6, results indicated that more severe dysphoria symptoms were associated with a lower delta activity to social feedback (*r* = −0.16, *p* < 0.05), but more severe social anxiety symptoms were associated with greater delta activity to social feedback (*r* = 0.16, *p* < 0.05). In contrast, neither dysphoria (*r* = 0.07, *ns*) nor social anxiety symptoms (*r* = −0.12, *ns*) were associated with delta activity for the monetary tasks.

**Figure 7:**
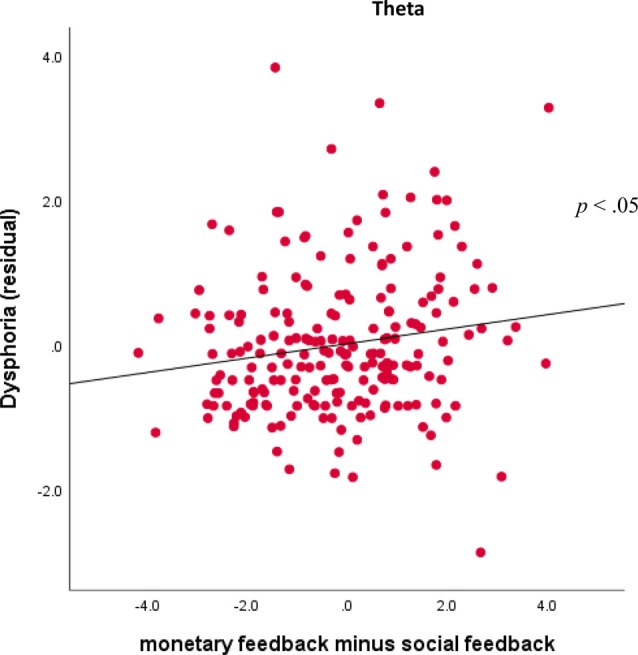
Associations between TF4SF1 (theta activity) for monetary feedback and symptoms. Scatterplots displaying the associations between theta activity for monetary feedback (averaged across valence and outcome) subtracting social feedback (averaged across valence and outcome) and dysphoria symptoms.

Finally, for the theta factor, results indicated a Domain × Dysphoria interaction, *F*_(1,201)_ = 5.29, *p* < 0.05, $\eta_{\text{p}}^{2}$ = 0.03. For follow-up analyses, theta activity was averaged across valence and outcome for monetary and social tasks. As shown in Figure 7, dysphoria was not significantly correlated with theta activity for monetary feedback (*r* = 0.01, *ns*) or social feedback (*r* = −0.10, *ns*) individually, but was correlated with monetary feedback minus social feedback (*r* = 0.16, *p* < 0.05).

### Domain Order

There was no effect of domain order on any of the three PCA factors (*p*s > 0.077). Therefore, no further ANCOVA analyses were conducted for the relationships with dysphoria and social anxiety.

## Discussion

The current study is the first to examine the time-frequency representation of electrocortical responses to monetary relative to social feedback. PCA of the time-frequency data in response to feedback revealed two delta factors and one theta factor. The delta factors were both modulated by combinations of task domain (monetary vs. social), valence (positive vs. negative), and outcome (correct vs. incorrect), and showed a general tendency of greater activity to rewarding feedback vs. non-rewarding feedback. In contrast, the theta factor was sensitive to outcome and valence, but not task domain. In addition, for the social domain, delta activity was greater to correct relative to incorrect feedback among those with more severe symptoms of anxiety, but smaller in those with more severe symptoms of depression. Overall, the current study demonstrates the importance of examining neural response to feedback processing *via* time-frequency analysis, especially in the context of the social domain.

PCA of the time-frequency data yielded two distinct delta factors, with one factor capturing lower frequency delta activity and the other factor capturing higher frequency delta activity. These two delta factors were similarly modulated by task effects such that both the intrinsic reward of being correct and positive valence feedback elicited higher delta activity overall across monetary and social domains. These results are consistent with previous findings of reward-related delta activity using monetary tasks (Bernat et al., 2011, 2015; Foti et al., 2011; Webb et al., 2017) and extend that research to the social domain, while also isolating effects associated with the intrinsic reward of being correct. It is important to note that the two delta factors also exhibited differences. Specifically, delta-low was sensitive to positive vs. negative valence and correct vs. incorrect feedback across domains. Delta-high showed greater activity to correct compared to incorrect feedback in both positive and negative social tasks, and in positive but not negative monetary task. In the negative monetary task (i.e., pick the door with the monetary loss), both outcomes were associated with potential conflict (e.g., losing money but being correct or breaking even but being incorrect), and this might explain why delta activity did not differ between the two outcomes. This was not the case for social feedback, suggesting that getting the correct social feedback was most salient. Overall, delta-high exhibited more nuanced task-manipulation effects compared to delta-low.

Unlike delta activity, theta activity was insensitive to task domain and was more sensitive to incorrect vs. correct outcome when the context was negative, and not to the monetary or social nature of the feedback. In experiments designed to elicit response errors, greater theta activity has been associated with error processing and conflict monitoring (Trujillo and Allen, 2007; Cavanagh et al., 2009; Cohen and Donner, 2013), and has been posited to be involved in increased cognitive control after committing errors (Cavanagh and Shackman, 2015). Our finding of greater theta activity to incorrect than correct feedback is hence consistent with these prior findings. On the other hand, previous studies using monetary gambling tasks have found theta activity sensitive to negative valenced outcome (i.e., loss; Bernat et al., 2015; Foti et al., 2015; Webb et al., 2017). However, under the current design, feedback of incorrect outcome and negative valence (i.e., no loss) compared to correct outcome negative valence (i.e., loss) was associated with greater theta activity, suggesting that theta activity may be more sensitive to outcome correctness than valence. In addition, this effect also applies to social feedback such that receiving incorrect feedback when guessing rejection elicited a higher theta than correctly guessing rejection.

The current findings are largely in line with previous studies showing more severe depression is associated with blunted reward-related delta in adults (Foti et al., 2015) and adolescents (Nelson et al., 2018), as well as greater loss-related theta (Webb et al., 2017). However, there are several unique aspects in the present study. First, none of these previous studies directly compared monetary vs. social tasks or correct vs. incorrect outcomes. The current study demonstrated that when these two variables were examined, the depression-related blunted delta was specific to social tasks and correct feedback, regardless of valence. This discrepancy indicates that being correct may be more salient than obtaining positive feedback and is more sensitive to individual differences in depressive symptoms. Additionally, in the sample of emerging young adults, delta activity to social feedback may be more sensitive to depression compared to monetary feedback. In terms of theta activity, there was no association between just monetary loss-related theta with depressive symptoms. Instead, it was the difference between monetary-related and social-related theta that was related to depression, regardless of valence or outcome. This difference from previous findings is possibly driven by critical task design differences mentioned above. Overall, these findings suggest that depression is related to blunted neural response to correct vs. incorrect social feedback and increased sensitivity to monetary compared to social feedback.

The current study is also the first to examine feedback-related delta and theta activity in association with social anxiety symptoms. Activity of both delta factors to social-feedback was associated with social anxiety symptoms. While lower depressive symptoms were associated with greater delta activity to social feedback, more severe social anxiety symptoms were associated with greater delta activity to social feedback. The positive association between social anxiety and social feedback-related delta activity suggests that individuals with more severe social anxiety show a greater difference in their delta activity in response to correct vs. incorrect social outcomes, regardless of the valence. This may indicate an increased sensitivity to being correct in making social judgments. Our findings suggest that, at least in non-clinical young adult samples, social feedback may be a more sensitive domain to elicit neural responses related to social anxiety symptoms compared to monetary feedback. These findings may underlie the neural processes contributing to the selective biases to negative social signals observed in individuals with social anxiety (Amin et al., 1998; Mogg et al., 2004).

Some limitations of the current study must be acknowledged. First, in order to control potential confounds, social feedback was purportedly provided by strangers. However, decision-making behaviors are sensitive to social feedback provided by a close friend but not strangers (Sip et al., 2015). Therefore, future research is needed to test whether the current results remain when relationship closeness is manipulated. Also, the monetary task involved equal wins and losses which may have negatively impacted participants’ motivation due to a lack of substantial incentives. Future studies are needed to examine the neural responses using unbalanced trials or manipulating the probability of winning vs. losing. Second, the study largely included young adults without clinically significant levels of anxiety and depression. Based on recommended cut-off scores from a recent study examining the clinical utility of IDAS-II scales (Stasik-O’Brien et al., 2019), 14 (~6.9%) and 20 (~9.8%) participants scored above the clinical cut-offs for depression and social anxiety, respectively. This limits the generalizability of the findings to other demographic populations and clinical samples. Furthermore, future research examining a broader range of socioeconomic status and age range (e.g., adolescence) are needed. Analytically, in order to keep it consistent with our previous investigation (Nelson et al., 2018) the current analyses utilized a baseline window that ended at the time of feedback onset, which can be suboptimal due to the potential temporal leakage of trial-related activity (Cohen, 2014). Future studies should consider the use of an earlier baseline period [e.g., −500 to −300 ms as previously suggested (Cohen, 2014)]. Also, the number of trials in the current study was based on prior psychometric research of time-domain RewP rather than time-frequency measurement. Future studies using a larger number of trials are encouraged to examine the replicability of the current findings. Finally, future research may examine whether the neural results probed by this laboratory experiment predict real-life decision-making both financially and socially. For instance, it remains to be tested whether blunted delta to social feedback and/or increased theta to monetary feedback predicts suboptimal decision-making in social networking and monetary investment.

In conclusion, the current findings suggest that previously demonstrated reward-related delta and non-reward-related theta activity are subject to the specific characteristics of feedback and outcome (e.g., domain, valence, and correctness). In addition, these results demonstrate the usage of time-frequency analyses to investigate dissociable neural processes in response to various aspects of feedback. This study also sheds light on the importance of examining neural responses to social feedback in understanding the neural processes in decision-making and elucidate their associations with psychopathology.

## Data Availability

All datasets generated for this study are included in the manuscript.

## Ethics Statement

All participants gave written informed consent and the study was approved by the Stony Brook University Institutional Review Board.

## Author Contributions

JMJ and BN contributed to task design and data collection. JJ, AS, and ZI conducted data analyses. JJ, AS, ZI, JMJ, and BN contributed to manuscript writing.

## Conflict of Interest Statement

The authors declare that the research was conducted in the absence of any commercial or financial relationships that could be construed as a potential conflict of interest.
